# Modeling the role of emotion regulation and critical thinking in immunity in higher education

**DOI:** 10.3389/fpsyg.2022.1005071

**Published:** 2022-09-29

**Authors:** Meilan Li, Tahereh Heydarnejad, Zeinab Azizi, Zeynab Rezaei Gashti

**Affiliations:** ^1^School of Overseas Education (School of Foreign Languages), Sanming University, Sanming, China; ^2^Department of English Language, Faculty of Literature and Humanities, University of Gonabad, Gonabad, Iran; ^3^Department of Teaching English and Linguistics, Faculty of Literature and Humanities, Ayatollah Borujerdi University, Borujerd, Iran; ^4^Department of Literature and Foreign Languages, University of Karaj, Karaj, Iran

**Keywords:** higher education, EFL University professors, critical thinking, emotion regulation, language teacher immunity, path analysis

## Abstract

It is deemed that the effectiveness of teachers is highly entangled with psycho-emotional constructs, such as critical thinking (CT), emotion regulation (ER), and immunity. Despite the potential roles of CR, ER, and immunity, their possible relationships have remained unexplored in the higher education context of Iran. To fill in this lacuna, this study explored the potential role of CT and ER in university teachers' immunity in the Iranian higher education context. For this purpose, a total of 293 English university teachers were selected using a convenience sampling method. They were invited to fill out the Watson–Glaser Critical Thinking Appraisal-Form, Language Teacher Emotion Regulation Inventory, and Language Teacher Immunity Instrument. The findings of path analysis indicated that the university teachers with higher CT were more productively immunized. Moreover, the results revealed that ER could predict the university teachers' immunity. The findings of the study lead to this implication that higher order thinking skills, emotion regulatory strategies, and immune enhancement should be incorporated into educational programs of higher education.

## Introduction

Clarifying the concept of effective teaching in both schools and higher education and conceptualizing the clear model of the effective teacher is not an easy task and is inherently contentious (Ericksen, 1984; Feldman, 1986; Brown and Atkin, 1988), since the term effective can be interpreted differently by different people and in a different context. Despite its long history, there is no agreed-upon definition for it. For instance, Hopkins et al. (1998) postulated three broad dimensions of effective teaching. The first dimension is teaching effects, a concept that reflects both teaching skills and teaching behaviors. The second relates to the acquisition of effective teaching models a teacher establishes in his/her classroom. The third dimension embraces teacher artistry, which highlights the teachers' responsibility for creating the conditions for effective learning. From Acheson and Gall's perspectives (Acheson and Gall, 2003), effective teaching involves the ability to provide instruction that creates an instructional climate that causes students to develop positive attitudes toward school and self (engaged and efficacious learners), helps students to develop the knowledge, skills, and understandings intended by curriculum objectives, and responds to initiatives for curriculum change so that the new curriculum's intents are fully realized. Burroughs et al. (2019) defined teacher effectiveness in terms of teacher experience, teacher professional knowledge, and teacher behaviors (p.8). Likewise, Elliott (2010) stipulated that teacher effectiveness is a combination of personality and ability, wherein the former is being regarded as a key factor (p.14).

Considering the pivotal role of effective teaching, Elliott (2010) identifies two subtopics related to teaching effectiveness: “effective teacher characteristics may be summarized as measuring who I am or the essence of teaching, whereas teacher effectiveness may be summarized as what I do or the process/product of teaching” (p. 1). From a social cognitive perspective, effective teachers are self-regulated individuals who take appropriate actions leading to the successful accomplishment of their professional tasks (Randi, 2004). In Feldman's perspective (Feldman, 1986), enthusiasm, positive self-regard, energy, and positive regard for others are the significant qualities of an effective teacher. In other words, the skills needed for effective teaching involve more than just expertise in an academic field. Effective teaching occurs best when teachers are empowered with desirable behavior and personality traits. Among several qualities and personality traits that are defined as the attributes of an effective teacher, ER, critical thinking (CT), and immunity, as well as their reciprocal relationships have remained uncharted territory in educational research, particularly in higher education. In addition, various challenges of the 21st century require more reflections on the contributing role of higher order thinking skills and self-aid constructs, fostering effective teaching.

Teaching bound with emotional experiences and teachers believe that regulating their emotions at the workplace leads to effective teaching (Sutton et al., 2009). Teacher ER refers to their abilities to manage and modify emotional experiences and expressions (Burić et al., 2017). ER empowers teachers to change the intensity and duration of their emotional experiences at the workplace (Chang and Taxer, 2020; Frenzel et al., 2021; Heydarnejad et al., 2021c), which have significant implications for manifesting teachers' effectiveness. Despite its relevance, and perhaps because of its complexity, teachers' ER, particularly English teachers' ER, is still in its infancy, and awaits further research (Burić et al., 2017; Alipour et al., 2021; Heydarnejad et al., 2021c). More specifically, Frenzel et al. (2015) asserted that teachers' emotions are different depending on different subjects and groups of students. Hence, each context is worth exploring as it may show different findings in comparison with other contexts.

As Chen and Cheng (2021) stipulated, handling emotionality and rationality as inevitable parts of teaching contribute effective teaching. Thereby, regarding the indisputable relevance of emotions and cognition at the workplace for teachers' effectiveness, it is important that teachers are armed with effective strategies and higher order thinking skills. CT as higher order thinking skills refers to analyzing and evaluating of the information through reflection and reasoning (Dewey, 1933; Paul, 1988). Through the lenz of CT, teachers think critically about their teaching strategies and look for evidence of effective teaching. It was evidenced that CT is associated with teachers' resilience (Ayoobiyan and Rashidi, 2021), self-regulation (Heydarnejad et al., 2021a), teaching style in higher education (Amirian et al., 2022), and professional identity (Sheybani and Miri, 2019). In addition, CT not only benefits individual university teachers but also the society as a whole.

The new born notion of language teacher immunity works as a defensive mechanism against different constraints in the realm of language teaching (Hiver and Dörnyei, 2017; Rahmati et al., 2019). Language teacher immunity can act as a shield to protect university teachers against high-intensity chaos and complexities of educational settings. What emerges from the review of the scare literature on language teacher immunity, it is positively correlated with teacher-related positive constructs (e.g., Hiver, 2017; Haseli Songhori et al., 2018; Rahimpour et al., 2020; Li, 2022). Yet, there is a dearth of literature about language teacher immunity, especially in higher education which echoes for more profound studies to investigate different aspects of language teacher immunity. To the best knowledge of the researcher, to date, no study has inspected these theoretically associated constructs within a single framework to disclose how they are linked with one another and consequently, how they affect teachers' job effectiveness. Therefore, more research is needed to fill this gap.

## Literature review

### Emotion regulation

The term emotion is derived from the Latin word “emovere”, which means to stimulate (Hargreaves, 1998). It means that the experienced emotions give direction to individuals' actions. To capture the concept of emotion, various definitions were posed based on different theoretical conceptualizations generated from physiology, philosophy, history, sociology, anthropology, and psychology (Hargreaves, 1998; Oatley, 2000; Frenzel, 2014; Burić et al., 2020; Uzuntiryaki-Kondakci et al., 2022). These conceptualizations share a common point in sense that ER is a complex, multi-component construct with different dimensions, namely, subjective, cognitive, motivational, expressive, and physiological (Lazarus, 2001; Scherer, 2009). Moreover, two outlooks can be defined for teachers' emotions: considering emotions as short-lived and relatively intense episodes or explaining them in a more trait-like manner or as relatively stable in time (Rosenberg, 1998). From a trait-like perspective, the average frequency of experienced emotions in teachers' professional lives is considered (Wood et al., 2008). In the current research, a trait-like manner is used to inspect university teachers' ER at their workplace.

Emotions are socially constructed phenomena that are uncovered in social interactions with others (Chahkandi et al., 2016). In other words, emotions derive their shape and meaning from the ideas and practices in the larger socio-cultural context (Boiger and Mesquita, 2012; Luque-Reca et al., 2022). The cultural context also plays a critical role in several aspects of individuals' emotional experiences. The ways of expressing and managing emotions are mostly consistent with the values, goals, and concerns in each cultural model. Interdependent cultures expect individuals to define themselves more in relation to others, prioritize harmony and interconnection, and try to adjust to each other's expectations (Chahkandi et al., 2016). Independent cultures, on the contrary, emphasize preserving individuals' autonomy through underlying individuals' uniqueness and self-esteem (Boiger and Mesquita, 2012; Ford and Mauss, 2015).

Additionally, cultures are not similar in the appraisals of the emotion-antecedent events (De Leersnyder et al., 2013). For instance, offensive situations are considered as threats to individual's autonomy and self-worth in North American contexts and asking individuals to cultivate high self-regard, assertiveness, and aggression. By contrast, offensive situations in Japanese contexts were interpreted as threats to social relationships and required individuals' understanding of the other persons' motives to be resolved (Chahkandi et al., 2016). Cultures also influence emotion display rules and individuals' motivation to exercise self-regulation (Ford and Mauss, 2015). That is, collectivist cultures (e.g., Asian American and Japanese contexts) tend to use ER more frequently and exert greater levels of emotion suppression than European American people (Gross et al., 2006). More specifically, cultures differ in the adaptation of ER strategies (Ford and Mauss, 2015). Cultures also are not similar in dealing with status and power relationships. Thus, they may expect the expression of emotions that maintain status and power and avoid emotions that threaten this differential (Matsumoto, 2006).

Teachers, in particular language teachers, experience various ups and downs at the workplace, which can trigger pleasant and unpleasant emotions. As Hargreaves (1998) put it, “emotions are at the heart of teaching” (p. 835). Teachers' emotional experiences affect their relationships with others (Richards, 2022), identity (Jones and Kessler, 2020), self-efficacy (Chen, 2018; Burić et al., 2020), pedagogical adoptions (Chen, 2020), work engagement (Burić and Macuka, 2017), as well as self-regulation, and teaching style in higher education (Heydarnejad et al., 2021a). Appraisal and attribution theories (Frenzel, 2014; Jacob et al., 2017; Frenzel et al., 2021) are the models used for explaining teachers' emotions. Appraisal theory is based on the indirect association between emotion and situation (Moors et al., 2013) and includes the following sub-sections: goal consistency, goal conduciveness, coping potential, goal attainment/impediment responsibility, and goal significance (Frenzel, 2014). Attribution is defined as a specific evaluation of the perceived causes of events (Jacob et al., 2017).

ER involves physiological, behavioral, and cognitive processes that each person utilizes to monitor, evaluate, and modulate their emotional experiences (Gross, 1998; Gross and John, 2003; Gross and Thompson, 2007). That is, ER acts as a campus and gives direction to individuals' emotions (Gross, 1998, 1999). The employed strategies in ER helps teachers to manage both pleasant and unpleasant emotions (Taxer and Gross, 2018). The activation of a regulatory goal, the engagement of regulatory processes, and the modulation of the emotion trajectory are the three core features of many diverse types of ER (Gross and Barrett, 2011). It is worth highlighting that ER activities may also happen explicitly or implicitly (Gross, 2014). In previous studies, explicit and implicit processes in ER are considered separately (Masters, 1991). However, it is recommended to consider ER processes as a continuum ranging from explicit, conscious, and controlled regulation to implicit, unconscious, effortless, and automatic regulation (Gyurak et al., 2011).

Theoretically, ER is supported by the process-oriented model of ER (Gross, 1998). The process-oriented model of ER is comprised of five temporal points (i.e., situation selection, situation modification, attention deployment, cognitive change, and response modulation). Recently, a model for the language teacher ER was proposed based on extensive review of the existing literature, the theoretical conceptualizations on ER in general, and teacher ER in particular (Heydarnejad et al., 2021c). This model involves six dimensions, i.e., situation selection, situation modification, attention deployment, reappraisal, suppression, and seeking social support. The three dimensions of situation selection, situation modification, and attention deployment were rooted in Gross's process-oriented model of ER (Gross, 1998). Reappraisal and suppression were based on Gross and John's conceptualization (Gross and John, 2003), and seeking social support as the last dimension was inspired by Jennings and Greenberg (2009) as well as Taxer and Gross (2018).

Research on university professor ER seems to be scarce. However, the conducted previous studies on teacher ER highlighted teacher-related variables, which affect or are affected by ER. As an example, Chang (2020) examined the relationship between teachers' beliefs about emotional display rules in the class, the attitudes toward ER strategies, and feelings of burnout. Based on the data analysis, display rules influenced expressive suppression and burnout. Moreover, the effect of cognitive reappraisals on teacher burnout was significantly negative. Results of this study emphasize that teacher education should be designed to help teachers to evaluate their beliefs about display rules and to involve in cognitive reappraisal. In another study, Morris and King (2018) investigated the role of emotion regulatory strategies in manipulating frustration among university language professors. Their findings suggested that university language professors employed ER strategies that increased their levels of confidence and helped overcome the stressors. The influence of ER strategies in return for classroom misbehavior in response to classroom misbehavior was explored by Chang and Taxer (2020). They found out that teachers who usually reappraise in the face of their learners' misbehavior are less probable to experience unpleasant emotions. Their findings show how teachers could regulate their negative emotions in the face of student misbehavior. By the same token, Fathi et al. (2021) explored the association between teacher reflection, self-efficacy, burnout, and ER among Iranian English teachers. The results of the structural model confirmed that ER would mediate the influences of teacher reflection and teacher self-efficacy on teachers' burnout among English teachers. In their conclusion, they offer some practical measures for teachers to monitor their emotional states.

### Critical thinking

CT was introduced by Socrates about 2 centuries ago, who maintained that assuming, questioning, reasoning, analyzing, and evaluating the inferences of individuals' activities are vital to justify their declarations (Fisher, 2001). Although CT has been applied in various territories (Philosophy, cognitive psychology, and education research), no unified definition was suggested for it (Thomas and Smoot, 1994; Solon, 2003). According to Halonen (1995), CT is mystified concept. Similarly, Fasko (2003) asserted that “there is no consensus on a definition of critical thinking” (p.8). From Dewey' perspective (Dewey, 1933), CT is active and regular evaluation of assumptions and suppositions to reach convenient inferences. Based on Paul (1988), CT is a higher order thinking skill, which involves analysis, syntheses, and evaluation. Furthermore, Halpern (2003) defined CT as the application of mental processes and cognitive skills, which foster the probability of desired behaviors.

From another viewpoint, Ennis (1996) defined CT as the intellectually disciplined process of actively and skillfully conceptualizing, synthesizing, and evaluating information generated by observation and reflection. Based on Thomas and Lok (2015), CT is formulated by knowledge, skills, and disposition. Moreover, Choy and Cheah (2009) defined teacher cognition through the lens of CT and concluded that these two constructs are integrated. More specifically, no concrete learning benchmarks are illustrated for CT progress (Stapleton, 2011). In spite of various proposed definitions and postulations, it is widely accepted that CT is a vital part of any successful education (Zhang et al., 2020; Heydarnejad et al., 2021a; Azizi et al., 2022).

Dewey (1933) was the first one who discussed about the importance of higher order thinking skills in education. He highlighted that reflective and CT skills must substitute the traditional ways of teaching, which concentrate on memorization and surface learning. As Davidson and Dunham (1997) argued, CT skills are teachable; thus, teachers a play significant role in teaching CT and developing critical minds (Bourdillon and Storey, 2002; Mason, 2008). In so doing, the teachers should learn how to think critically. About the crucial role of CT and its enhancement, Zhang et al. (2020) have conducted a study among English university teachers to gauge their attitudes toward CT and its applications in their teaching. As their findings revealed, English university teachers confirmed that CT should be an integral part of classroom teaching. In addition, it was also concluded in another recent study that CT and self-regulation give directions to teachers' preferred teaching styles (Heydarnejad et al., 2021b; Parveen et al., 2022).

Furthermore, it was approved that CT influenced teachers' professional identity (Sheybani and Miri, 2019). In this regard, Jenkins (1998) asserted that CT skills broaden teacher competencies and help them to build greater autonomy at work. The contributions of teachers' metacognitive skills, academic self-efficacy, and their CT skills is supported by Kozikoglu and Babacan's findings (Kozikoglu and Babacan, 2019). They highlighted the need for more research to understand how higher order thinking skills can be practiced among teachers. Taken a similar path, Sadeghi et al. (2020) sought to inspect qualitatively the constructs of CT from viewpoints of the English teachers and learners. Based on data analysis, they suggested some strategies for reinforcing CT ability such as: Discussion, group working, Interpretation, Open-mindedness, self-awareness, to name a few. In this study, pedagogical implications for English teachers were suggested to practice CT skills among their learners. They also invited curriculum developers and syllabus designers to consider CT activities in teaching materials and support in-service classes for teachers. Although the role of teachers' CT in their progress at work and implementing CT in their students is approved by different empirical studies, some teachers still used rote learning. It is of great importance to engage learners at schools as well as universities to ponder on challenging questions and make inferences (Sadeghi et al., 2020; Heydarnejad et al., 2021b; Rezai et al., 2022). The nature of CT, teachers' lack of knowledge and experience, as well as their inabilities in fostering CT skills may be among the possible reasons for not applying CT in the major parts of teaching (Buskist and Irons, 2008).

### Teacher immunity

Stemmed from the Latin word “immunis”, teachers' immunity is a recently introduced concept to language teaching discipline (Hiver and Dörnyei, 2017). Biologically, immunity is defined as a protective system that activates naturally occurring antibodies and plays down infection through biochemical reactions (Janeway et al., 2005). It works as a defensive system that fights against pernicious, undesirable, or detrimental effects of the external environment (Hiver, 2015). Similarly, teacher immunity refers to a defensive and adaptive mechanism, which works against various conflicts and challenges at the workplace (Hiver, 2015, 2017). As Hiver and Dörnyei (2017) stipulated, teacher immunity is an amalgamation of motivation to teach, psychological wellbeing, and openness to change on one end and teaching pressures, burnout, and attrition on the other end of the spectrum.

The formation of teacher immunity is based on self-organization theory that is adapted from complexity theory (Larsen-Freeman, 2012; Sampson, 2022). Self-organization refers to a process through which the complete function of a dynamic system alters through the interaction of different parts of that system (Larsen-Freeman, 2012; Gooran et al., 2022) and includes four developmental stages: triggering, coupling, realignment, and stabilization (Rahmati et al., 2019). Similar to its origin in biology, teacher immunity is of two kinds: productive immunity and maladaptive immunity (Hiver and Dörnyei, 2017; Sutarto et al., 2022). As a protective armor, the former protects teachers against stress, failure, burnout, and the like. In contrast, the latter negatively affects the teaching processes to make them fossilized (Hiver and Dörnyei, 2017). Different factors may trigger maladaptive immunity, such as avoidance-oriented behaviors (Hiver and Dörnyei, 2017) or resistance to change or innovation (Bullough and Hall-Kenyon, 2012; Xu et al., 2022). Productive immunity influences teachers' thinking style, acting in social contexts, as well as professional identity (Hiver, 2017; Hiver and Dörnyei, 2017). More precisely, language teacher immunity can be classified as productive immunity, maladaptive immunity, the state of immunocompromised, and partial immunity. Productive immunity refers to a vigorous form of teacher immunity, while maladaptive immunity is the counterproductive form of teacher immunity. Immunocompromised means having not developed any coherent form of teacher immunity, and partially immunized refers to halfway features of teacher immunity.

What emerges from the review of the sparse literature on teacher immunity, this road is untrodden and calls for further studies to shed light on its associations with other teacher-related constructs. After the introduction of language teacher immunity by Hiver (2015, 2017) and (Haseli Songhori et al., 2018), the dominant type of employed immunity strategy was investigated among Iranian English teachers by Haseli Songhori et al. (2018). They found out maladaptive immunity was the predominant type of immunity among Iranian English teachers. Furthermore, they concluded that Iranian English teachers followed triggering, coupling, realignment, and stabilization, in forming their immunity. In the same vein, Rahimpour et al. (2020) applied a path-analysis approach and postulated a model on the factors predicting language teacher immunity. Based on their findings, language teacher immunity is indirectly influenced by agreeableness, extroversion, and emotionality through job insecurity and reflective teaching. They also concluded that the influence of job insecurity on reflective teaching and language teacher immunity was significantly negative.

Along the same path, the relationship between autonomy, emotions, engagement, and immunity of experienced in-service teachers was investigated by Azari Noughabi et al. (2020). As the results of multiple regression suggested, language teacher immunity could be significantly predicted by teachers' autonomy, emotions, and engagement. Among the three variables under study, teacher autonomy was found to be the strongest predictor of experienced EFL teachers' immunity. The implications of this study ask for providing EFL teachers with opportunities to exercise autonomy and regulate emotions through teacher education courses, which in turn foster productive immunity. Moreover, the contributions of L2 grit and work engagement to EFL teachers' immunity examined (Azari Noughabi et al., 2022). Their findings reflected those higher levels of work engagement and L2 grit immune EFL teachers in the face of different challenges during their professional lives. In a recent study in China, Li (2022) concluded that the relationship between EFL teachers' immunity, mindfulness, and work engagement was significantly positive. This study also necessitates the use of training courses for language teachers to enhance EFL teachers' immunity development, mindfulness, and engagement.

### Objectives of the present study

In spite of its relevance, and perhaps because of its complexity, teachers' ER and immunity, in particular English university teachers' ER and immunity has remained an uncharted territory that awaits further research (Burić et al., 2017; Hiver and Dörnyei, 2017; Rahimpour et al., 2020; Alipour et al., 2021; Heydarnejad et al., 2021c). More importantly, Frenzel et al. (2015) asserted that teachers' emotions are different depending on different subjects and groups of students. Hence, each context is worth exploring as it may show different findings in comparison with other contexts. Most of the existing studies on teachers' ER has been conducted within a theoretical framework of stress and coping (Lewis and Haviland, 1993) or in the context of emotional labor (e.g., Hargreaves, 1998, Isenbarger and Zembylas, 2006; Azari Noughabi et al., 2020, 2022). Regarding teacher immunity, few empirical studies (Hiver, 2015, 2017; Haseli Songhori et al., 2018; Rahimpour et al., 2020) and only one theoretical study (Hiver and Dörnyei, 2017) have been conducted among language teachers. Thereby, the realm of higher education still remained untouched and calls for more identical studies that put forward a clear picture of university professor immunity.

**Figure 1:**
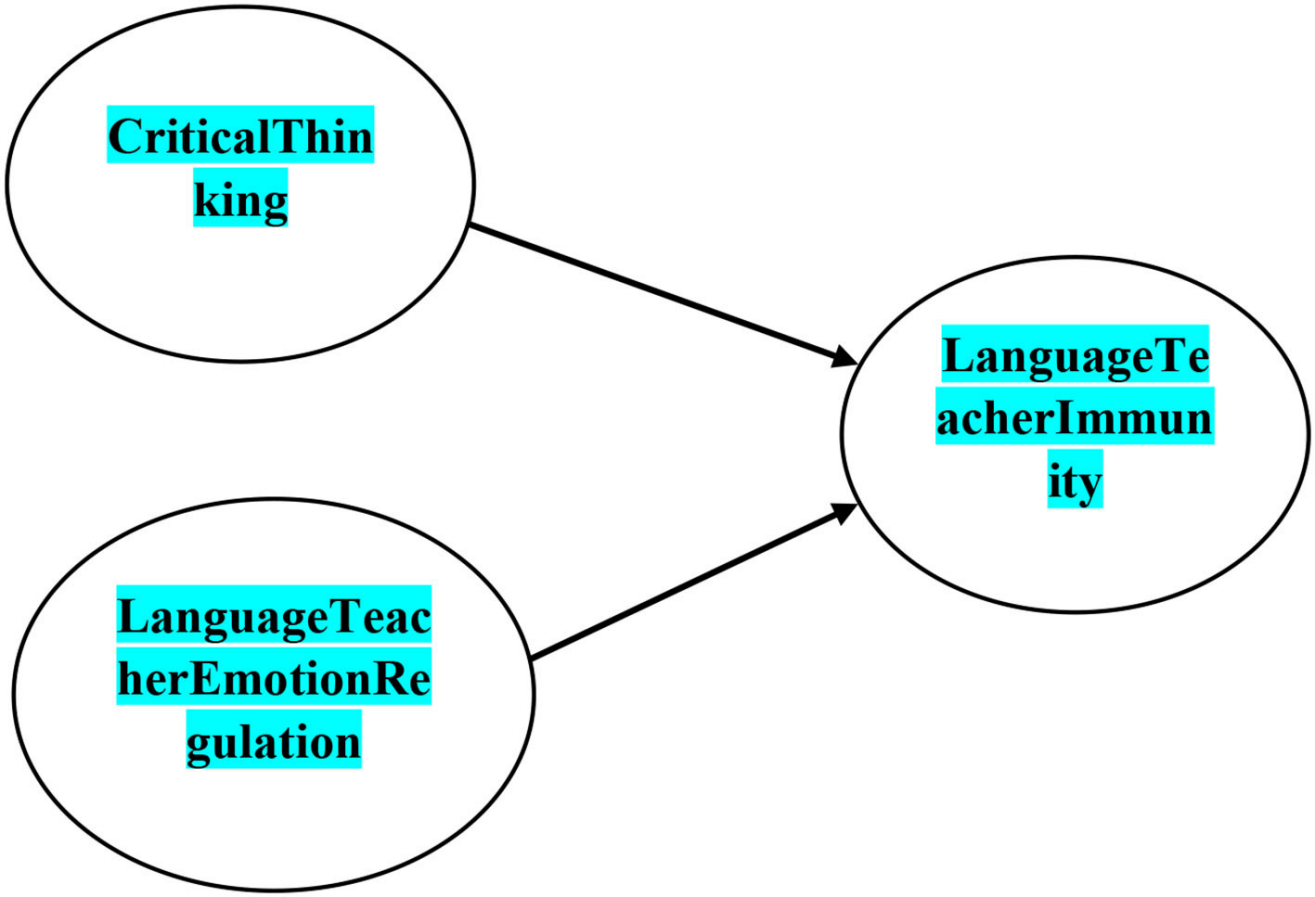
Theoretical structural equation model.

Furthermore, it is generally accepted that CT has numerous benefits for teachers, but little is known about how it interacts with two other essential constructs, i.e., language teacher ER and immunity, especially in higher education. Leafing through the existing literature reflects that the possible relationship between ER, CT, and immunity has not been brought to the foreground of research foci (Gross and Thompson, 2007; Burić et al., 2017; Rahimpour et al., 2020; Sadeghi et al., 2020; Li, 2022), particularly in higher education (Fathi and Derakhshan, 2019; Chang, 2020; Chang and Taxer, 2020; Heydarnejad et al., 2021a; Amirian et al., 2022). To this end, the present study sought to propose a model to display the contribution of CT as well as ER to immunity in higher education (see Figure 1). Considering the abovementioned objectives, the current investigation put forward to answer the following research questions:

RQ1: To what extend does English university teachers' critical thinking predict their immunity?
RQ2: To what extend does English university teachers' emotion regulation predict their immunity?

In line with the above research questions, the following null hypotheses were formulated:

H01. English university teachers' CT does not predict their immunity.
H02. English university' teachers' emotion regulation does not predict their immunity.

### Theoretical model

The present study is built on the assumption that university teachers' immunity is affected by CT and ER. That is, it is hypothesized that university teachers' immunity is shaped with their CT and ER.

## Method of the study

### Research design

The researchers employed a correlational design for the present study. As noted by Riazi (2016), a correlational design is used to explore the correlations between some variables without controlling or manipulating any of them. Overall, the researchers used a correlational design to uncover the role of CT and ER in university teachers' immunity in the Iranian higher education context.

### Setting and participants

The present study was run at state-run universities in Iran. They are under the direct supervision of Ministry of Science, Research and Technology. The primary mission is to guarantee free education and physical training for everyone at all levels, and the facilitation and expansion of higher education. Using a convenience sampling method, a total of 293 English university teachers were selected from 25 run-state universities. According to Riazi (2016), the convenience sampling method is a non-probability sampling method adopted by researchers to gather data from a conveniently available pool of participants. They included both men (*n* = 171) and women (*n* = 122) aged from 31 to 52. They had different majors, including English Teaching (*n* = 110), English Literature (*n* = 74), English Translation (*n* = 61), and Linguistics (*n* = 48). Due to logistical limitations, the participants' years of teaching and teaching location were not controlled. Of particular note is that the participants declared their consent to participate in the study orally. The researchers ensured that their responses would be kept confidential and they would be kept informed about the final results.

### Instruments

#### Watson–Glaser critical thinking appraisal-form

The Watson**–**Glaser Critical Thinking Appraisal Form (1980) was the applied instrument to explore CT among the participants. This instrument was generated from Watson and Glaser (1980) and includes the following sections: inference, recognizing of assumptions, making deduction, interpretation, and evaluation. In a study conducted by Watson and Glaser (2002), the scale presented acceptable validity and reliability. In the present study, Cronbach Alpha was 0.944, which indicated acceptable reliability.

#### The language teacher emotion regulation inventory

The Language Teacher Emotion Regulation Inventory (LTERI), designed and validated by Heydarnejad et al. (2021c), was employed to gauge university teachers' ER strategies. They were required to consider similar situations from their teaching experiences at the workplace and rate the statements in the light of their preferred ER strategies. The LTERI consists of 27 items on a five-point Likert scale anchored by 1 (“never”) and 5 (“always”) with six components, i.e., situation selection (5 items), situation modification (5 items), attention deployment (4 items), reappraisal (5 items), suppression (4 items), and seeking social support (4 items). The reliability for all sub-scales of the LTERI was acceptable (ranging from 0.718 to 0.814) in a study by Heydarnejad et al. (2021c). In the current study, the reliability of the LTERI estimated through Cronbach's alpha was acceptable (ranging from 0.735 to 0.932).

#### The language teacher immunity instrument

To measure the participants' immunity, the Language Teacher Immunity Instrument (LTII), designed and validated by Hiver (2017), was utilized. This instrument is composed of 39 items in 7 sub-scales, each with a 6-point response scale (1 = strongly disagree; 6 = strongly agree). The sub-scales of this instrument are as follows: Teaching self-efficacy (7 items), Burnout (5 items), Resilience *(*5 items), Attitudes toward teaching (5 items), Openness to change (6 items), Classroom affectivity (6 items), and Coping (5 items). In the current investigation, the reliability of the LTII estimated through Cronbach Alpha was acceptable (ranging from 0.831 to 0.948).

### Data collection procedures

The participants were selected based on convenience or opportunity sampling procedures, and they were assured that their responses were entirely anonymous. A web-based platform was employed to conduct this investigation, which was started in January and ended in June 2022. That is, the participants received an electronic survey form including Watson–Glaser Critical Thinking Appraisal-Form A, the Language Teacher Emotion Regulation Inventory (LTERI), and The Language Teacher Immunity Instrument (LTII) through Google Forms. Since all teachers were qualified enough in English, the language of all four scales was English and, in this way, a construct irrelevant factor was avoided. Conducting the electronic survey enables researchers to collect data from different regions with varying age groups and teaching experiences. Altogether 293 forms were received with an 87.2% return rate. Moreover, no data were missed due to the design of the electronic survey.

### Data analysis procedures

As the first step, the reliability of the instruments was checked by Cronbach Alpha formula. Then, the normality distributions of the data were checked through the Kolmogorov-Smirnov Test. Further, descriptive statistics were used to describe the data. Finally, as the data were normally distributed, confirmatory factor analysis (CFA) and structural equation modeling (SEM) using LISREL 8.80 were employed to analyze the data. That is, all latent variables were validated using CFA before testing a structural model (Hair et al., 1998). SEM as a robust multivariate procedure was used to take a confirmatory hypothesis-testing approach for the proposed structural theory (Schreiber et al., 2006).

## Results

The results of statistical analysis to probe into the relationship between CT, ER, and immunity were presented here. Table 1 reported the descriptive statistics of English university teachers' CT, ER, and immunity.

**Table 1:** The results of descriptive statistics of the english university teachers' critical thinking, emotion regulation, and immunity.

**Inventory**	**N**	**Minimum**	**Maximum**	**Mean**	**Std. deviation**
Inference	293	1.00	5.00	3.874	0.854
Recognizing of assumptions	293	1.00	5.00	3.646	0.726
Making deduction	293	1.00	5.00	3.715	0.440
Interpretation	293	1.00	5.00	3.636	0.619
Evaluation	293	1.00	5.00	3.735	0.678
Situation selection	293	1.20	5.00	3.666	0.890
Situation modification	293	1.00	5.00	3.853	1.011
Attention deployment	293	1.00	5.00	3.928	0.653
Reappraisal	293	1.00	5.00	3.512	0.714
Suppression	293	1.00	5.00	2.555	0.643
Seeking social support	293	1.00	5.00	3.921	0.818
Teaching self-efficacy	293	1.00	6.00	4.632	0.493
Burnout	293	1.00	5.80	2.451	0.764
Resilience	293	1.00	5.60	4.543	0.643
Attitudes toward teaching	293	1.00	5.86	4.623	0.518
Openness to change	293	1.17	6.00	4.187	0.495
Classroom affectivity	293	1.00	6.00	3.996	1.051
Coping	293	1.00	6.00	4.807	1.091

As Table 1 presented, among the CT subscales inference (M = 3.874, SD = 0.854) and evaluation (M = 3.735, SD = 0.678) got the highest mean scores. Regarding the Language Teacher Emotion Regulation subscales, attention deployment (M = 3.928, SD = 0.653) and seeking social support (M = 3.921, SD = 0.818) show the highest mean scores. Moreover, among the Language Teacher Immunity subscales, coping (M = 4.807, SD = 1.091) and teaching self-efficacy (M = 4.632, SD = 0.493) displayed the highest mean scores.

As the following step, the data distributions were examined to make a logical decision about applying appropriate statistical methods. To do so, the Kolmogorov–Smirnov test was used to check the normality distributions of the variables.

Based on Table 2, the sig value for all the scales and their subscales was higher than 0.05, which the data were normally distributed. Thus, parametric methods could be employed for testing the related research hypotheses. The LISREL 8.80 statistical package was applied to explore the structural relations among the variables in the present research.

**Table 2:** The results of kolmogorov–smirnov test.

**Inventory**	**Subscales**	**Kolmogorov–** **Smirnov** **Z**	**Asymp. Sig.** **(2-tailed)**
Watson–Glaser critical thinking appraisal	Inference	0.689	0.729
	Recognizing of assumptions	0.737	0.649
	Making deduction	0.707	0.699
	Interpretation	1.081	0.193
	Evaluation	0.796	0.551
LTERI	Situation selection	0.711	0.694
	Situation modification	0.705	0.702
	Attention deployment	0.687	0.733
	Reappraisal	0.817	0.517
	Suppression	1.082	0.192
	Seeking social support	1.054	0.217
LTII	Teaching self-efficacy	0.891	0.405
	Burnout	0.602	0.862
	Resilience	0.895	0.399
	Attitudes toward teaching	0.907	0.383
	Openness to change	1.186	0.120
	Classroom affectivity	0.980	0.292
	Coping	0.872	0.432

The chi-square magnitude, the root-mean-square error of Approximation (RMSEA), the comparative fit index (CFI), and the normed fit index (NFI) were utilized to evaluate the model fit. As Jöreskog (1990) stated the chi-square should be non-significant and the chi-square/df ratio should be lower than 3. Furthermore, the root-mean-square error of approximation (RMSEA) is suggested to be lower than 0.1 (Jöreskog, 1990). The NFI with the cut value greater than 0.90, GFI with the cut value greater than 0.90, and CFI with the cut value greater than 0.90 indicates a good fit (Jöreskog, 1990). As Table 3 reported, the chi-square/df ratio (2.593) and the RMSEA (0.074) were also acceptable. The other three fit indices, GFI (0.938), NFI (0.944), and CFI (0.925) reached the acceptable fit thresholds.

**Table 3:** The results of fit indices (model 1).

**Model**	**Cut value**	
χ2		342.28
df		132
χ2/*df*		2.593
RMSEA	>0.1	0.074
GFI	0.9<	0.938
NFI	0.9<	0.944
CFI	0.9<	0.925

**Figure 2:**
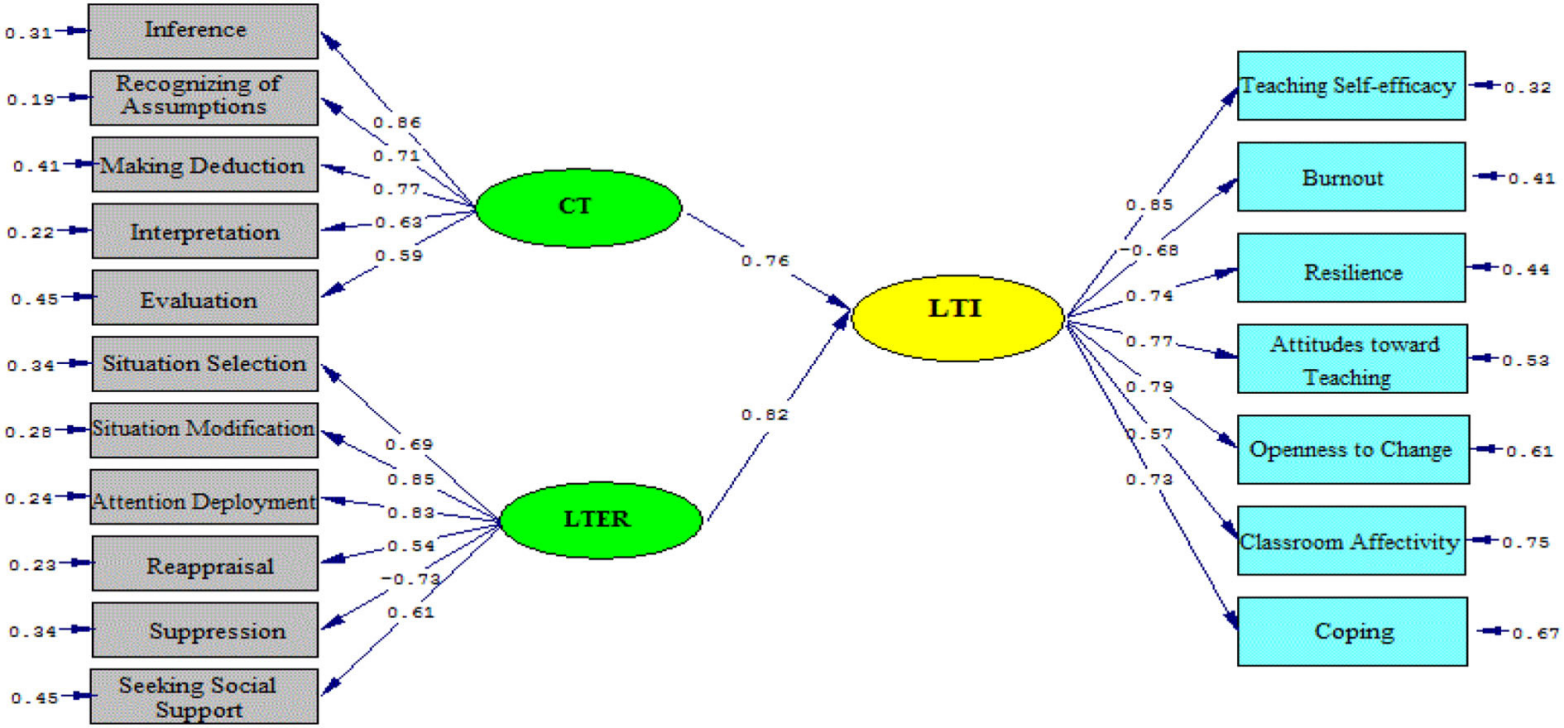
Schematic representation of path coefficient values for the relationships between critical thinking, emotion regulation, and immunity (model 1).

**Figure 3:**
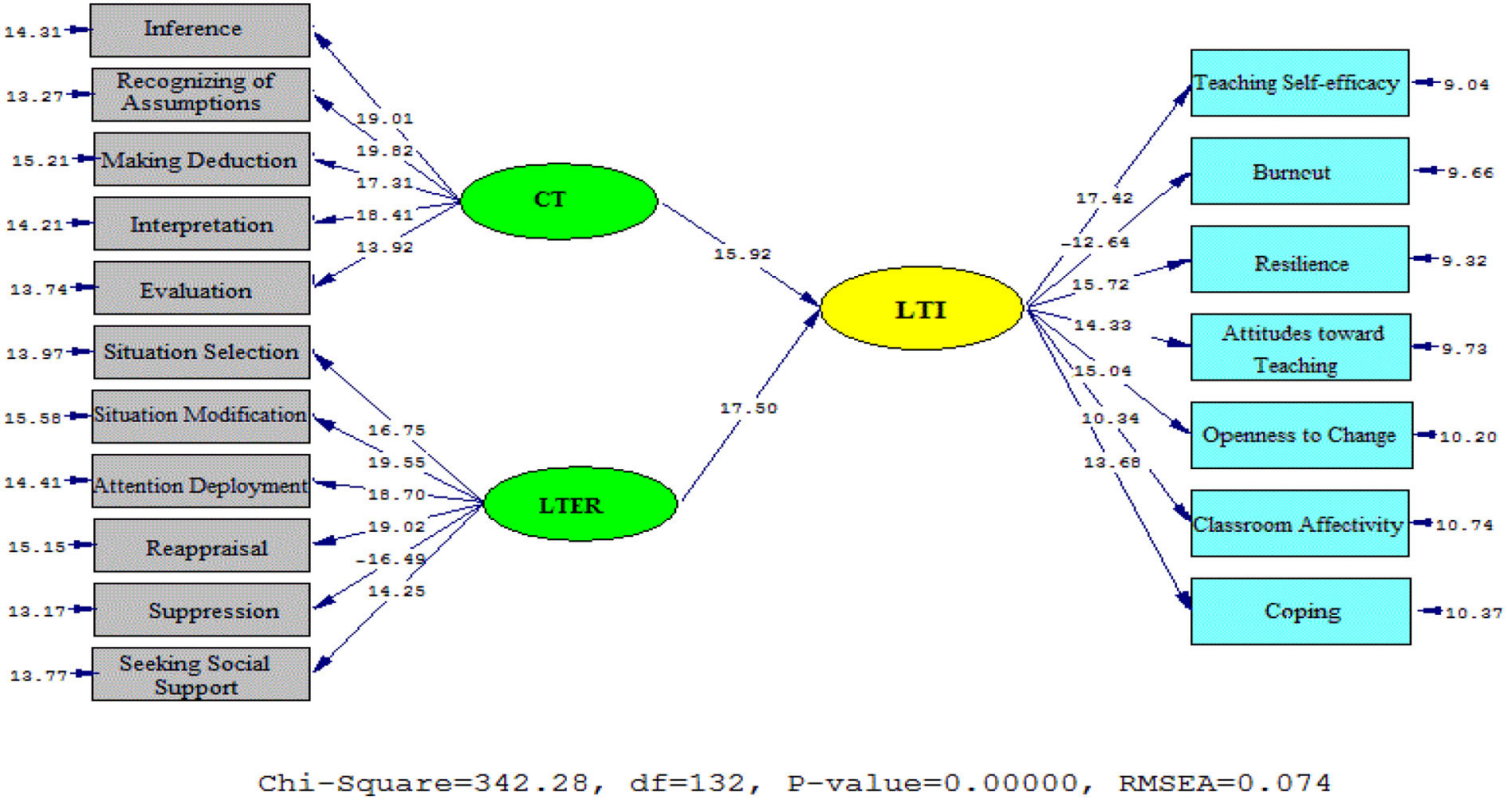
The T values for path coefficient significance (model 1).

As Figures 2, 3 (model 1) illustrated, the impacts of CT and LTER on LTI were positive. That means, CT significantly and positively contributed to the English university teachers' immunity (β = 0.76, t = 15.92). The significant role of university professor ER on teacher immunity (β = 0.82, t = 17.50) was also reported.

**Figure 4:**
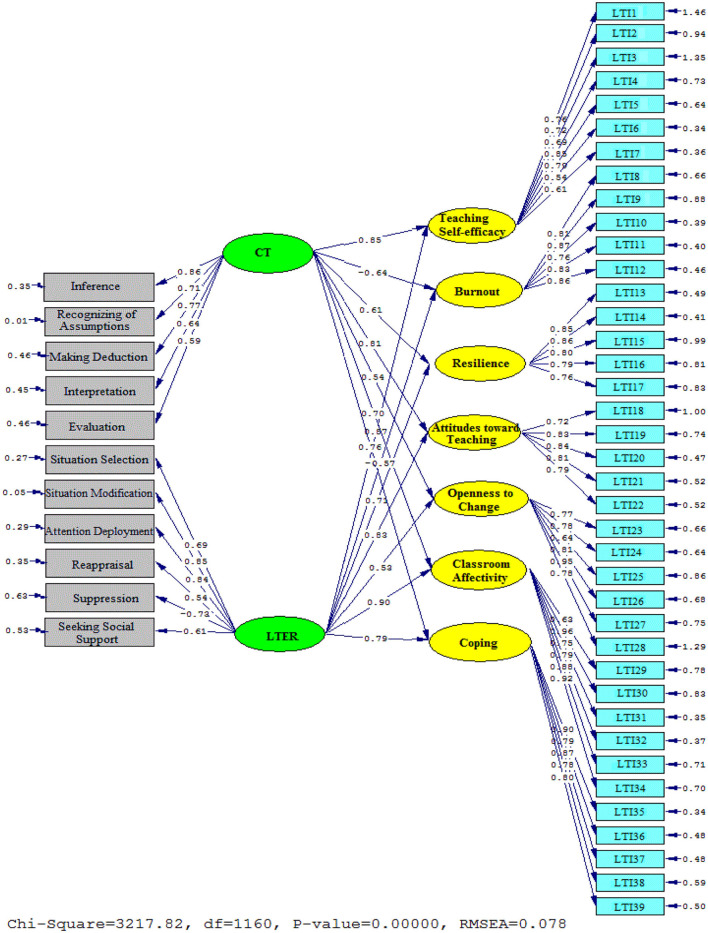
Schematic representation of path coefficient values for the influential role of critical thinking and self-efficacy on teaching style' subscales (model 2).

Table 4 presented the chi-square/df ratio (2.773), the RMSEA (0.078), GFI (0.932), NFI (0.941), and CFI (0.955) related to the second model. Based on the Table 4, all of the fit indices got the acceptable fit thresholds. The following figures (Figures 3, 4) depicted the detailed relationships among the variables.

**Table 4:** The results of fit indices (model 2).

**Model**	**Cut value**	
χ2		3217.82
df		1160
χ2/*df*		2.773
RMSEA	>0.1	0.078
GFI	0.9<	0.932
NFI	0.9<	0.941
CFI	0.9<	0.955

**Figure 5:**
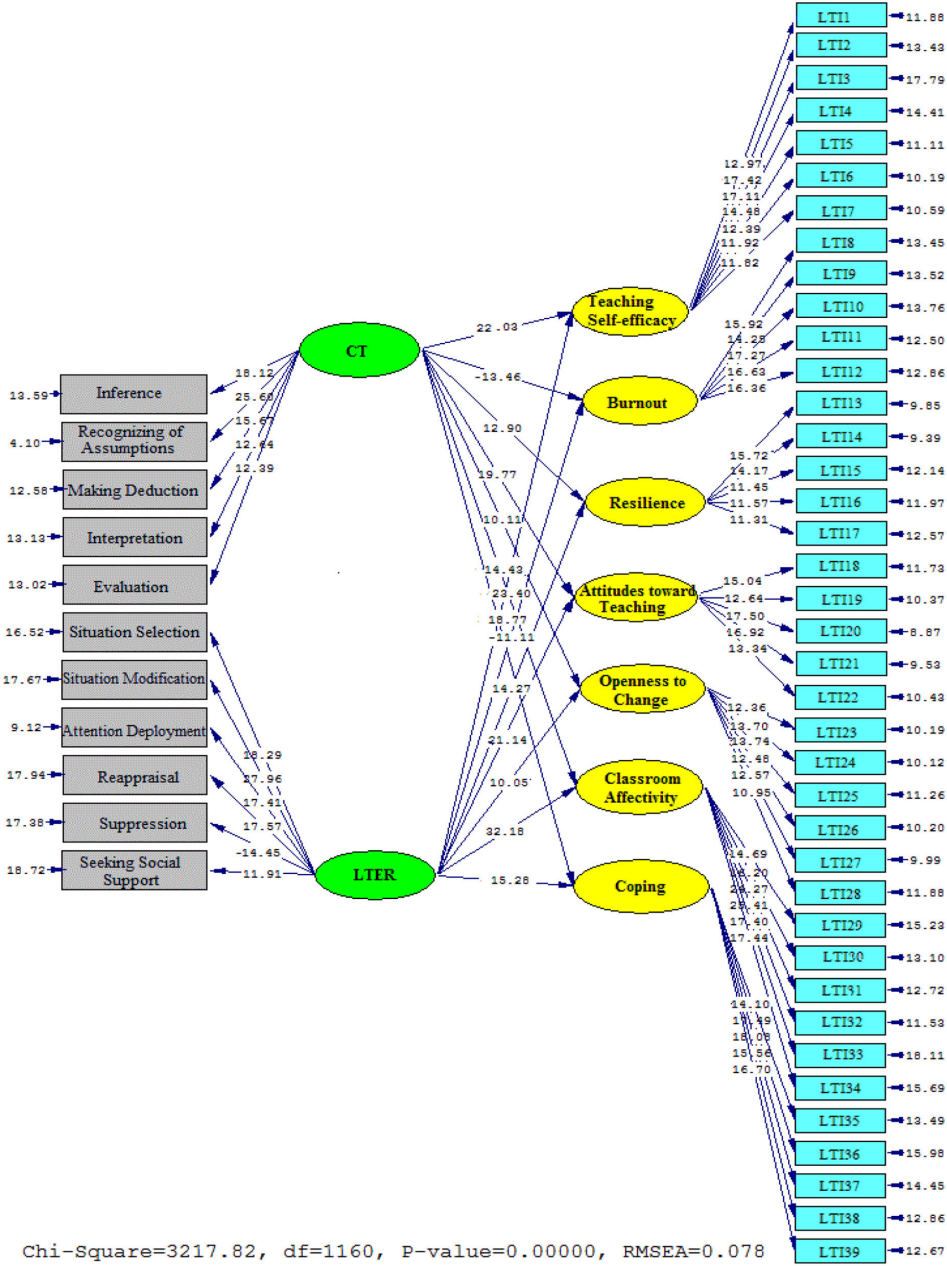
The T values for path coefficient significance (model 2).

Figures 4, 5 portray the contributions of CT and LTER to LTI sub-components. As it was depicted, CT significantly and positively contributed to the LTI sub-components: Teaching self-efficacy (β = 0.85, t = 22.03), Resilience (β = 0.61, t = 12.90), Attitudes toward teaching (β = 0.81, t = 19.77), Openness to change (β = 0.54, t = 10.11), Classroom Affectivity (β = 0.70, t = 14.43), and Coping (β = 0.76, t = 18.77). By contrast, the contribution of CT on Burnout (β = −0.64, t = −13.46) was significantly negative.

Considering the contribution of LTER to LTI subcomponents, the results was as follows: Teaching self-efficacy (β = 0.87, t = 23.40), Resilience (β = 0.71, t = 14.27), Attitudes toward teaching (β = 0.83, t = 21.14), Openness to change (β = 0.53, t = 10.05), Classroom Affectivity (β = 0.90, t = 32.18), and Coping (β = 0.79, t = 15.28). In contrast, the contribution of LTER to Burnout (β = −0.57, t = −11.11) was significantly negative.

Table 5 displayed that CT correlated positively with LTII subcomponents as following: Teaching self-efficacy (r = 0.874, *p* < 0.0.01), Resilience (r = 0.717, *p* < 0.01), Attitudes toward teaching (r = 0.852, *p* < 0.01), Openness to change (r = 0.562, *p* < 0.01), Classroom Affectivity (r = 0.723, *p* < 0.01), and Coping (r = 0.814, *p* < 0.01). In contrast, the association between CT and Burnout was negative (r = −0.679, *p* < 0.01). Moreover, about the relationships between LTER and LTII subcomponents, the results were as follows: significantly positive with Teaching self-efficacy (r = 0.895, *p* < 0.01), Resilience (r = 0.773, *p* < 0.01), Attitudes toward teaching (r = 0.895, *p* < 0.01), Openness to change (r = 0.598, *p* < 0.01), Classroom Affectivity (r = 0.945, *p* < 0.01), and Coping (r = 0.845, *p* < 0.01); significantly negative with Burnout (r = −0.589, *p* < 0.01).

**Table 5:** The results of the correlation coefficients among the english university teachers' critical thinking, emotion regulation, and immunity.

	**Critical thinking**	**LTERI**	**Teaching** **self-efficacy**	**Burnout**	**Resilience**	**Attitudes** **toward** **teaching **	**Openness to** **change **	**Classroom** **affectivity **	**Coping**
**Critical thinking**	**1**								
Language Teachers Emotional Regulation Inventory	0.621^**^	1							
Teaching self-efficacy	0.874^**^	0.895^**^	1						
Burnout	−0.679^**^	−0.589^**^	−0.644^**^	1					
Resilience	0.717^**^	0.773^**^	0.677^**^	−0.756^**^	1				
Attitudes toward teaching	0.852^**^	0.895^**^	0.773^**^	−0.607^**^	0.646^**^	1			
Openness to change	0.562^**^	0.598^**^	0.650^**^	−0.443^**^	0.564^**^	0.705^**^	1		
Classroom affectivity	0.723^**^	0.945^**^	0.881^**^	−0.664^**^	0.611^**^	0.452^**^	0.467^**^	1	
Coping	0.814^**^	0.845^**^	0.740^**^	−0.720^**^	0.658^**^	0.624^**^	0.555^**^	0.645^**^	1

** Correlation is significant at the 0.01 level (2-tailed).

## Discussion

This study explored the possible role of CT and ER in English university teachers' immunity in higher education. The results evidenced that CT is a strong predictor of the English university teachers' immunity. Based on the findings, it may be argued that CT empowers the English university teachers to zoom on their teaching processes leading to higher immunity. In other words, aligned with the findings of the study, it may be argued that the participants who were critical in their profession, they might have gained a comprehensive understanding the planning, implementing, and evaluating of the teaching processes. This, in turn, might have empowered them to overcome the tensions and unpleasant situations in their job. Accordingly, the first null hypothesis stating that the English university teachers' CT does not predict their immunity was rejected. The contribution of higher order thinking skills to English teachers' immunity has been confirmed in the previous studies (e.g., Rahmati et al., 2019; Rahimpour et al., 2020; Atefi Boroujeni et al., 2021; Li, 2022). For instance, Rahimpour et al. (2020) found that reflective teaching and language teachers' immunity were closely related. The gained findings also corroborated with those of Rahmati et al. (2019), emphasizing the cultivation of reflection in developing language teachers' immunity. Furthermore, the obtained results offered a deeper picture of how the English university teachers' CT may predict the different subskills of immunity. As the findings depicted, the participants' CT had significant positive correlation with their self-efficacy, resilience, attitude toward teaching, coping, openness to change, and classroom affectivity. In line with the findings, it may be argued that critical analysis of teaching experiences might have affected the English teachers' self-efficacy beliefs and led to positive attitudes toward their job. The results are consistent with those of Amirian et al. (2022) highlighting the strong correlation between higher order thinking skills and self-efficacy. The relationship between the English teachers' resilience and metacognitive skills was also confirmed by Mehrabian et al. (2022). The association between self-efficacy, resilience, and burnout was also supported by the findings of Fathi and Saeedian (2020). In the same line of inquiry, the link between CT and openness to change as one of the sub-components of personality trait was concluded by Acevedo and Chelsie (2022), as well as Frenzel et al. (2021). One possible reason for the gained findings is that the English teachers who were highly reflective in their job, they might have applied coping strategies result in the promotion of perseverance and productive immunization. Another justification for the findings may is that being equipped with CT might have enabled the English university teachers to show remarkable resilience in the face of tensions and difficulties.

Moreover, another line of discussion for the gained findings may be ascribed to view that the university teachers who were more critical thinkers might have managed reappraisal tends and coped with the challenges and difficulties of their jobs (Pe et al., 2013; Sheppes et al., 2014). In other words, along with the gained results, it may be argued that CT might be a way to immunize the English university teachers productively in the face of tensions and complexities of the working conditions. In support of this argument, Wang et al. (2022) found the interrelationships of teacher higher order thinking skills, positive emotions, and resilience. Additionally, the findings receive support from the past literature disclosing the noticeable contributions of CT to the efficiency of English teachers (e.g., Sheybani and Miri, 2019; Sadeghi et al., 2020; Heydarnejad et al., 2021a; Amirian et al., 2022). Furthermore, a part of the findings documented that the relationships between the reflective teaching and burnout were significantly negative. That is, it may be argued that that the more the English teachers might evaluate their teaching processes, the less chronic stress, emotional exhaustion, feeling of ineffectiveness, and lack of accomplishment they might experience. These findings are consistent with those of the previous studies (e.g., Khodabakhshzadeh et al., 2017; Li et al., 2021), indicating a negative relationship between CT and teacher burnout.

Additionally, the results documented that ER was highly correlated with the English university teachers' immunity. Thus, the second null hypothesis stating that the English university teachers' ER does not predict immunity was rejected. In other words, the findings documented that ER had positive and significant contributions to self-efficacy, resilience, attitude toward teaching, coping, openness to change, and classroom affectivity (the sub-scales of teacher immunity). Additionally, the results demonstrated that the regulation of the English university teachers' emotions at the workplace decreased the likeliness of burnout. Along with Wang et al. (2022), it can be argued that psychological wellbeing might lead to a productive configuration of immunity among the English university teachers. In a same vein, Hiver (2017) argued that the emotional wellbeing of English teachers would guarantee the development of productive immunity. The findings of the study are in line with those of Burić et al. (2020), reporting that teachers' emotions performed as a filter governing the way efficacy information is interpreted. Additionally, the results are congruent with the findings of Donker et al. (2020). They found that strong ER strategies played a significant role in decreasing teachers' emotional exhaustion and burn out. Furthermore, the gained findings lend support to those of Shen (2022), disclosing the mediator role of teacher ER in managing teachers' burnout, stress, and anxiety among English teachers.

One possible explanation for the findings may be ascribed to the view that the emotion-regulatory strategies might endow a balance in the professional lives of the English university teachers, leading to more enthusiasm and engagement in teaching procedures. Additionally, the findings may be justified from this perspective that ER might contribute to the latency, rise time, magnitude, duration, and offset of emotional responses and immunize university teachers productively. The findings of the current study can be strongly supported by the underpinning theories of CT, ER, as well as immunity. CT stipulated that higher order thinking skills offer stages of conceptualization, analysis, synthesize, reflection, and evaluation (Dewey, 1933; Paul, 1988). Productive immunity stemmed in self-organization theory is a defensive mechanism act against different experienced problems during the professional life (Larsen-Freeman, 2012; Hiver, 2015, 2017). This rational can be put forward that the strategies involved in higher order thinking skill support self-awareness and self-organization lead to productive immunity. Moreover, the model of teacher ER suggests skillful teachers adapt efficient strategies in managing their emotions (Heydarnejad et al., 2021c). Emotional balance, which is the results of self-evaluation and self-organization fosters productive immunity. In other words, cultivating emotional regulation keeps university teachers' immune system productive. Reciprocally, optimizing immune competence among university teachers fosters efficient instruction (Hiver and Dörnyei, 2017).

In addition, it can be argued that CT skills and ER might help the English university teachers to achieve a balance in their personal and professional lives. That is, this rationale can be put forward that thinking and evaluation allow university teachers to delve into their behaviors and activities, giving them a strong sense of self-awareness, self-regulation, self-monitoring, and self-assessment in the face of emotional experiences in their personal and professional demands. Furthermore, it can be implied that the more teachers practice reflective teaching, the better they can manage and modify their emotional demands. This finding is congruent with prior studies though limited and quite rare in the EFL context, which focus on the relationship between reflective teaching and teacher emotions (Zembylas, 2014; Bleakley et al., 2020; Gkonou et al., 2020; Song, 2021).

## Conclusion

As noted above, the present study explored the role of the English teachers' CT and ER in immunity. The findings revealed that the English teachers' CT and ER contributed significantly to immunity. That is, the English university teachers armed with CT skills and ER strategies might manipulate their practices and align them with the emotional display rules of their profession. This implied that the English university teachers were immunized with CT and ER to handle job obligations.

The implications drawn from the results of the current study may be beneficial for teacher-educators to develop more productive pre-service and in-service programs by incorporating CT and ER in their syllabi. Additionally, teacher preparation programs should consider more practical strategies to enhance CT skills, ER strategies, and higher order thinking skills for pre-service teachers. Considering the centrality of university teachers' affective status in how they deal with reform initiatives, it is hoped that the outcomes of this research help university teachers take practical measures to monitor and manage their emotional states in English education in Iran and in the broader international context. Besides, policymakers are invited to consider these results in order to have a comprehensive picture of factors that contribute to the success and failure of teachers and programs. Since language teacher immunity is relatively a new construct, educators, teachers, and policymakers need to become aware of its central role in the field. Therefore, studies like the present investigation provide useful insights for those involved in the language teaching profession.

Some limitations imposed on the present study that can be considered as avenues for further research. First, as the participants were chosen through a convenience sampling method, more studies should be conducted in other higher education contexts in the country to increase the generalizability of the obtained findings. Second, as a quantitative method was applied in this study, future studies can use mixed-methods designs to inspect the association between CT, ER, and immunity to present a comprehensive picture of the topic. Third, because the present study was cross-sectional, future longitudinal studies are needed to inspect the long-term contributions of CT and ER to university teachers' immunity. Fourth, because in the present study, demographic variables such as teachers' cultural and socioeconomic background, major, mastery experience, pedagogical training, and other possible explaining variables were not explored. Thus, researchers are recommended to consider university teachers' demographic variables in similar research studies in the future. Fifth, studies conducted within the realm of educational psychology indicated that the performance of the participants with different L1 backgrounds might differ considerably from culture to culture and that the methodological approaches to measure this issue in specific contexts might not be comparable. Therefore, the relationships between ER, CT, and immunity can be the target of future research in other contexts and cultures. Sixth, it is recommended to undertake further research to explore the possible contributions of university teachers' CT tendencies, ER, and immunity to their learners' academic achievement. As further suggestion, examining the relationships between ER, CT, and immunity with other teacher attributed constructs, such as autonomy, reflective teaching, self-regulation, L2 grit, and work engagement, are recommended. Last but not least, as the present study focused on the role of CT and ER in university teachers' immunity, interested researchers can explore the correlation between teachers' immunity and their job motivation, job satisfaction, and job performance.

## Data availability statement

The original contributions presented in the study are included in the article/supplementary material, further inquiries can be directed to the corresponding author/s.

## Author contributions

All authors listed have made a substantial, direct, and intellectual contribution to the work and approved it for publication.

## Funding

This paper was supported by the Construction of Critical Reading, a provincial first-class offline undergraduate course, by Fujian Provincial Department of Education in 2021 (Code No. 323).

## Conflict of interest

The authors declare that the research was conducted in the absence of any commercial or financial relationships that could be construed as a potential conflict of interest.

## Publisher's note

All claims expressed in this article are solely those of the authors and do not necessarily represent those of their affiliated organizations, or those of the publisher, the editors and the reviewers. Any product that may be evaluated in this article, or claim that may be made by its manufacturer, is not guaranteed or endorsed by the publisher.
